# Targeting monoamine oxidases for the treatment of betel quid dependence: a potential pharmacological and neurobiological approach

**DOI:** 10.3389/fphar.2026.1853911

**Published:** 2026-07-03

**Authors:** Sorbomita Chakraborty, Abdul Hussain Choudhury, Indu Sharma, Yashmin Choudhury

**Affiliations:** 1 Department of Biotechnology, Assam University, Silchar, India; 2 Department of Microbiology, Assam University, Silchar, India

**Keywords:** addiction, betel nut (BN), betel quid (BQ), cessation, monoamine oxidase (MAO), tobacco

## Abstract

The trend of betel quid chewing has witnessed a gradual upsurge in recent years, indicating increased dependence on betel quid/betel-nut and complications of withdrawal. Dedicated efforts towards the development of effective cessation strategies are thus the need of the hour. This review examines the complex relationship between dependence on betel quid with or without tobacco and the activity of monoamine oxidase A, emphasizing a critical yet underexplored area in betel quid addiction. We have analyzed emerging evidence suggesting that arecoline, betel quid’s primary psychoactive constituent, functions as a monoamine oxidase A inhibitor, likely modulating dopaminergic and serotonergic pathways involved in addiction and mood regulation. This inhibitory effect may be pronounced in users who combine betel quid with tobacco, yielding synergistic neurochemical alterations that may enhance dependency. Current treatment approaches largely hinge on behavioral and educational strategies, with minimal pharmacological options available. We propose that understanding the role of monoamine oxidase A in betel quid addiction could pave the way to understanding the complexities of betel quid dependence, especially when consumed with tobacco, and monoamine oxidase A inhibition by the controlled administration of pharmacological inhibitors may serve as a potential strategy for treating withdrawal symptoms and dependence. Future research should include investigating specific monoamine oxidase A expression patterns in betel quid users, developing culturally. Appropriate interventions that integrate biological awareness, and exploring the need for carefully timed monoamine oxidase A inhibitors as cessation aids.

## 1 Introduction

Betel quid (BQ) is an edible substance or a blend of ingredients typically containing areca nut popularly known as betel nut (BN) with or without tobacco, and other flavouring agents such as catechu, cardamom or cloves, in raw, processed, or manufactured form (IARC, 2004). Some variations also include tobacco (such as *sadaguda, khaini, zarda, pan masala*), creating a more potent mix (IARC, 2012). BN is the fourth most addictive substance globally, and its use is inextricably linked with smokeless tobacco (SLT) use, posing a serious public health concern especially in South East Asia and the Pacific regions, where this combination is prevalent, and driven by traditional practices as well as high dependence rates (Shao et al., 2026). The Asian–Pacific region is the epicenter of the BQ/BN chewing habit where it is responsible for a significant burden of cancer, especially oral and esophageal cancers. The prevalence of BQ abuse, dependence, and Betel Quid Use Disorder (BUD) varies widely by region, *viz.* South Asia (0.8%–46.3%), Southeast Asia (0.4%–43.5%), and East Asia (4.7%–39.2%). In certain populations, the severity of these issues reaches as high as 99.6% for abuse, dependence, and BUD (Ko et al., 2024). Furthermore, a study among the Micronesian population in Guam revealed threefold higher incidence of oral cancer among individuals who chew BQ with tobacco compared to non-chewers. Salivary biomarkers, such as N-nitrosoguvacoline and 4-(methylnitrosamino)-1-(3-pyridyl)-1-butanone (NNK), were found to spike during dual use (Franke et al., 2015). This study was also supported by a study in Myanmar where 84% of the users combined BQ with tobacco which significantly increased cancer risk, particularly oral and throat cancers. Diseases such as, oral submucous fibrosis, leukoplakia, and oral squamous cell carcinoma were also reported by some of the users (Papke et al., 2020a).

Variations in methods of preparation of BQ products taken with tobacco lead to differences in pH, resulting in higher nicotine absorption and increased addiction potential (Thawal et al., 2023). Based on the present global trends, people who use processed BN preparations were found to have higher levels of addiction and cravings than people who use traditional BN preparations (Gupta and Jain, 2024). Alarmingly, a recent study in India shows a high incidence of BQ/BN usage (16%) among pregnant women. Besides, BN/tobacco-based products have been aggressively marketed and advertised in small, visually enticing, and affordable packets, leading to a substantial surge in sales since early 1980s. As a result of these strategies, a considerable number of school going children are attracted to these products and begin consumption of BN in different forms at an early age. This trend has raised concerns regarding the potential health consequences associated with early exposure and habitual use of this substance among young individuals (Wazir et al., 2017).

Different countries have implemented different approaches to control the use of tobacco and BQ/BN. Most clinical intervention for BQ/BN dependence have adapted treatments from nicotine replacement therapy (NRT), cytisine and varenicline, with a success rate of 20% (Papke et al., 2020b), which may not address the complex neurochemical responses arising from use of BQ/BN use with or without tobacco. Current international intervention strategies emphasize behavioral modifications and educational awareness, while pharmacological treatments remain limited. The World Health Organization (WHO) recommends integrating BN nut cessation into tobacco dependence treatment, with strategies like clinic-based programs and mobile health technologies (mHealth) like SMS support, for rural areas (Mehrtash et al., 2017). Some progress has been achieved through regulatory measures, such as Taiwan’s sales restrictions and India’s gutkha prohibition, which have contributed to reduced consumption among urban youth (Chu, 2001; Dhingra and Jhanjee, 2023).

While educational and behavioural interventions are beneficial to some extent, pharmacological approaches for BQ cessation remain limited and largely unapproved. Recent neuroimaging and biochemical studies have revealed that chronic BQ/BN users have altered neurotransmitter systems, including, dopamine, serotonin and norepinephrine. Arecoline, the primary psychoactive alkaloid of BN has been shown to possess monoamine oxidase A (*MAO-A)* inhibitor-like properties, which increases neurotransmitter concentrations in the brain (Hung et al., 2024; Ko et al., 2020). These findings directly link BQ with *MAO* enzymes which metabolize neurotransmitters in the brain’s synapse.

Thus, the prevalence of BN/BQ use and prevailing cessation strategies exhibit a counterproductive effect and the issue is likely to persist if not addressed urgently (Gupta and Jain, 2024). This review is focussed on targeting *MAO-A* as a potential strategy for treating BQ addiction. The objective of the review is to unveil the underlying neurochemical mechanisms involving *MAO-A* activity in the development of BQ dependence*,* which can be used to identify potential cessation strategies through examining the bidirectional relationship between arecoline and *MAO-A* exposure. The review will also discuss how modulation of *MAO-A* may affect BQ reinforcement, mitigate withdrawal symptoms, and potentially serve as a novel pharmacological target for BQ dependence.

## 2 Methodology

A narrative review was conducted using articles published up to 4 June 2026. These articles were retrieved from PubMed and Google Scholar (Cals and Kotz, 2016) using the following combinations of keywords and MeSH terms (National Library of Medicine, 2020) were used: (i) addiction and neuropharmacology, (ii) arecoline and mAChRs, (iii) arecoline and nAChRs, (iv) arecoline and nicotine, (v) betel nut components and metabolic pathways, (vi) betel quid and betel nut, (vii) betel quid and its preparation (viii) monoamine oxidase (*MAO-A* and *MAO-B*), (ix) natural *MAO* inhibitors, (x) prevalence and epidemiology, (xi) treatment and cessation interventions. Representative terms included “addiction,” “alkaloids,” “anthraquinones,” “arecoline,” “arecoline pathway,” “betel nut,” “betel nut components,” “betel quid,” “bromophenols,” “cessation,” “chromenones,” “coumarins,” “dependence,” “fatty acids,” “flavonoids,” “*MAO*-A,” “*MAO* inhibitors,” “*MAO* inhibitors in betel nut,” “*MAO* inhibitors in smoking and smokeless tobacco,” “*MAO* gene structure,” “mAChR,” “monoamine oxidase,” “nAChR,” “phenols,” “polyphenols,” “prevalence,” “saponins,” “sterols,” “tannins,” “terpenoids,” “treatment,” and “xanthones.”

The inclusion criteria were original research articles, review articles, meta-analyses, and clinical studies published in peer-reviewed journals on betel quid, betel nut, arecoline, monoamine oxidases, addiction pathways (arecoline and nicotine in smoking and smokeless tobacco), *MAO* inhibitors in smoking and smokeless tobacco, prevalence, epidemiology, cessation, and interventions. Human, animal, and *in vitro* studies published in English were considered. Duplicate publications, and studies focusing exclusively on oral cancer, smoking tobacco, alcohol use, or other substance use disorders without relevant neuropharmacological, epidemiological, or treatment-related data, were excluded.

The retrieved articles were reviewed and screened for relevance based on their titles, abstracts, and full texts by SC, AHC and YC. Data were extracted based on BQ prevalence estimates, BQ components and their effects, *MAO* activity and genetic variation, arecoline pharmacology and metabolism, neurotransmitter alterations, addiction mechanisms, natural *MAO* inhibitors, and treatment outcomes. Articles unrelated to the study objectives were excluded. Publications that did not address the specific topics of the current review were removed during this stage (Roychoudhury et al., 2021).

## 3 Arecoline and BQ addiction

Despite awareness of the adverse effects of BQ/BN chewing, many users have a difficult time quitting due to the addictive properties of BN which are attributed to the alkaloids, *viz.*, arecoline, betelidine, guvacoline, and nicotine present in it. The varying levels of these alkaloids may influence the degree of addiction (Chen et al., 2021). Arecoline in particular, deeply penetrates the brain, where it exerts parasympathetic and muscarinic effects, contributing to the addiction and frequent consumption of BN. Arecoline binds to nicotinic acetylcholine receptors (nAChR) contributing to the addictive properties of BN and stimulating the sympathetic nervous system. Studies indicate that arecoline’s effects peaks within 4–6 min, with elevated concentrations persisting in the mouth for up to 10 min after chewing begins. This suggests that the active compounds released during BN chewing are primarily absorbed through the mucous membranes of the oral cavity (Chen et al., 2022).

**FIGURE 1:**
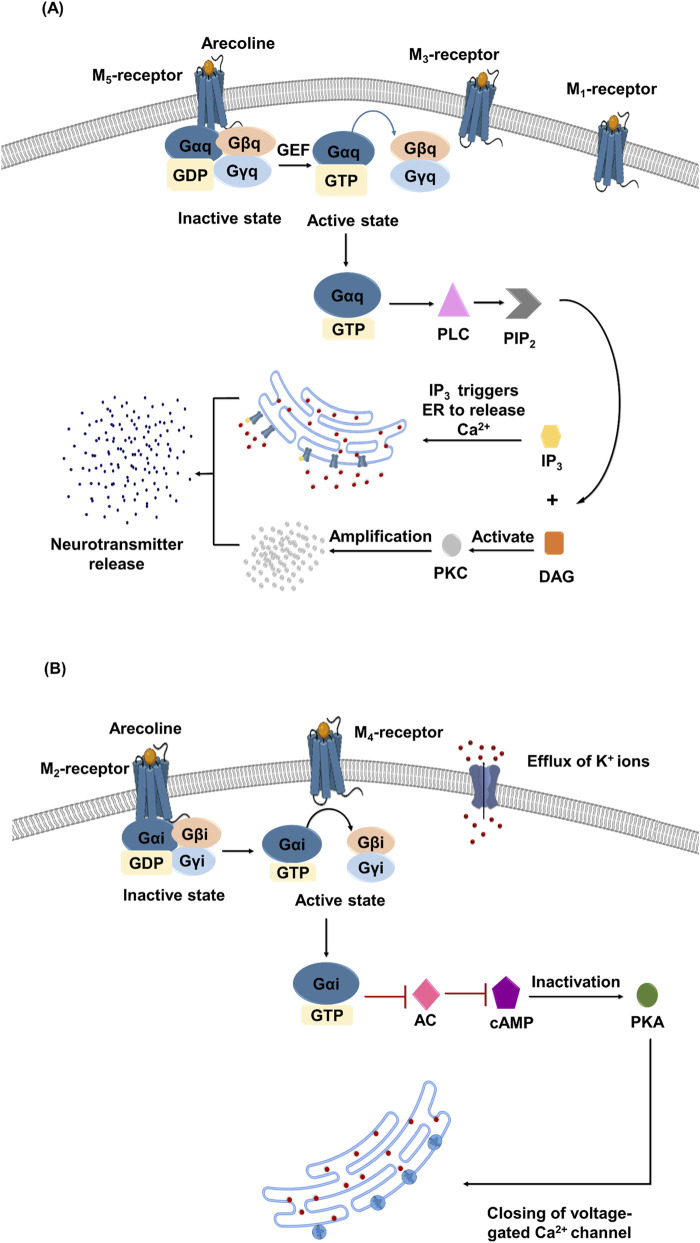
Arecoline and mAChR pathway **(A)** Excitatory pathway-Arecoline binds to M_5_ muscarinic acetylcholine receptors on the neuronal membrane causing, a conformational change in the receptor. These receptors are connected to heterotrimeric G-proteins (Gαq, Gβq and, Gγq) in their GDP-bound state. When the receptor activates, it acts as a guanine nucleotide exchange factor (GEF), swapping GDP for GTP. The activated Gq protein stimulates phospholipase C (PLC), which in turn hydrolyzes phosphatidylinositol 4,5-bisphosphate (PIP_2_) into inositol triphosphate (IP_3_) and diacylglycerol (DAG). IP_3_ increases intracellular Ca^2+^ release from the endoplasmic reticulum (ER) while DAG activates protein kinase C (PKC) to phosphorylate proteins and regulate neuronal activity. Elevated Ca^2+^ level and PKC activity enhance the release of excitatory neurotransmitters, such as dopamine. **(B)** Inhibitory pathway-When arecoline binds to M_2_ or M_4_ receptors, it activates the Gi protein. The activated Gi protein inhibits adenyl cyclase (AC). This decreases cyclic AMP (cAMP) levels, which lowers the activation of protein kinase A (PKA) and reduces the phosphorylation of ion channels. Thus, M2 and M4 activation leads to the opening of K^+^ channels and closing of Ca^2+^ Channels, causing neuronal hyperpolarization and reduced neurotransmitter release. Figure elements courtesy of Biorender (www.biorender.com) and Scidraw (www.scidraw.io); all remaining figures were created independently in Microsoft PowerPoint.

Arecoline is cholinomimetic and has an analogous binding pattern to acetylcholine (Locock et al., 2016), binding to orthosteric site of M5 muscarinic acetylcholine receptors (mAChR) receptor. This causes conformational change in the receptor as the transmembrane helix VI (TMVI) swings outward. The outward movement of TMVI creates a space where Gq-protein can dock onto the receptor. This triggers the Gq-protein, hence activates downstream signaling, like stimulation of phospholipase C (PLC), production of inositol triphosphate (IP_3_), Ca^2+^ release, and dopamine regulation (Eglen and Nahorski, 2000) (Figure 1).

The stimulation of M5 receptors by arecoline increases dopamine transmission in the neural reward pathways within the brain, especially in the dorsal striatum and nucleus accumbens (NAc) (Figure 1). This mechanism affects how brain circuits adapt and alter. Arecoline, like nicotine, increases impulses to dopaminergic (DAergic) neurons, which contributes to its addictive properties. It also affects smooth muscle activity and interacts with γ-amino butyric acid (GABA) receptors in the brain, resulting in psychedelic effects. The DAergic neurons are important in learning, reward, and addiction. They are modulated by both GABAergic and cholinergic (nicotinic and muscarinic) inputs. The primary reward pathway in the brain where dopamine levels rise, is comprised of the prefrontal cortex (PFC), NAc, and ventral tegmental area (VTA) (Sariah et al., 2019).

Long-term drug use, such as BQ use alters brain structure, neuron function, and connectivity over time, creating strong memories associated with drug use, and eventually disrupting this dopamine reward system. People who are addicted frequently have fewer D2 receptors, which reduces their sensitivity to the reward system in the brain. This decrease could help to explain the obsessive drive to continue using drugs in order to experience the pleasure they once did. The PFC normally aids in habit control and lessens the desire for rewards; however, addiction impairs this function, resulting in impulsive actions and an increased need to seek substances. This explains the significant dependence exhibited by a large proportion of BQ users (Sariah et al., 2019).

## 4 Arecoline and nicotine: a shared pathway

**FIGURE 2:**
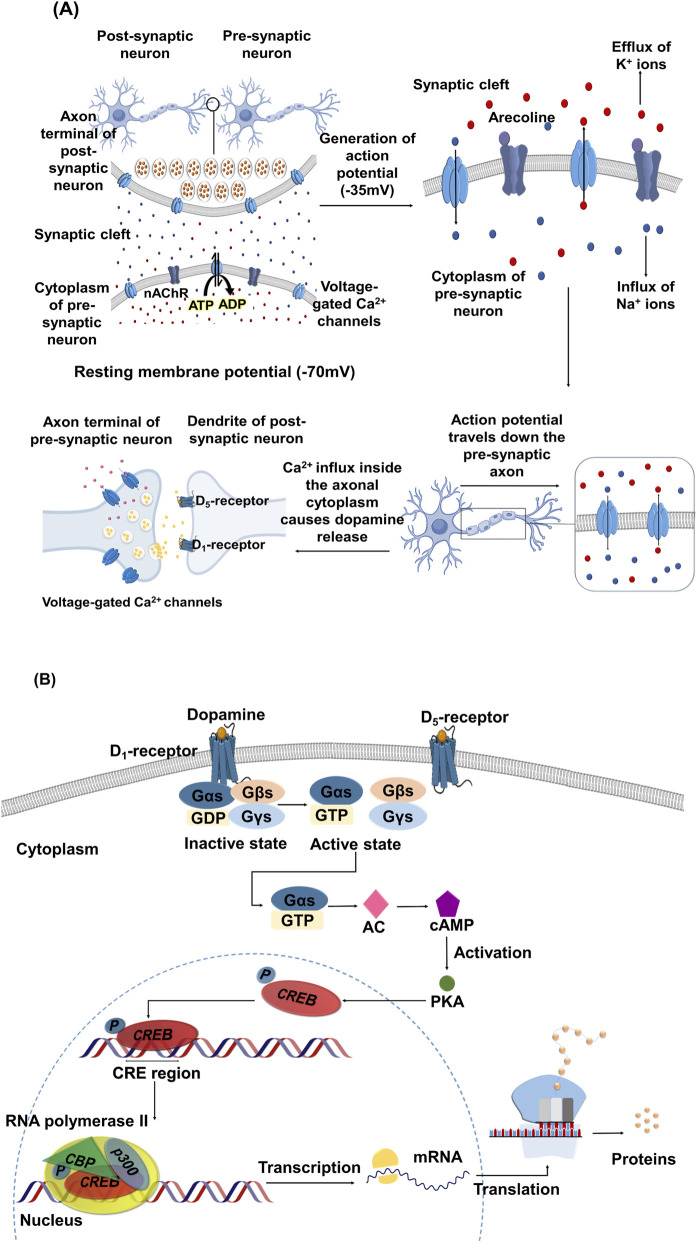
Arecoline’s pathway via nAChR **(A)** The resting membrane potential (−70 mV) is maintained by Na^+^/K^+^ ATPase pump (3 Na^+^ out and 2 K^+^ in). Arecoline binds to α4β2* subtype of nAChRs of the pre-synaptic neuron (dendrites), which causes changes in receptor conformation, opening the ion channels. This causes membrane depolarization (−35 mV) by influx of Na^+^ ions and efflux of K^+^ ions. The action potential generated travels down the pre-synaptic axon which is followed by opening of voltage-gated Ca^2+^ channel, allowing Ca^2+^ influx from the extracellular fluid. Ca^2+^ binds to synaptotagmin, activating SNARE proteins (such as synaptobrevin, SNAP-25, syntaxin). This causes synaptic vesicles fuse with the presynaptic membrane and release neurotransmitters, such as dopamine and serotonin, in the synaptic cleft (exocytosis). **(B)** Dopamine binds to dopamine receptors (D_1_ and D_5_) in the post-synaptic neuron. This binding causes conformational changes in the receptor and activates Gs proteins by exchanging GDP for GTP. The activated Gs-protein activates adenyl cyclase (AC) which in turn activates cyclic AMP (cAMP). The cAMP then activates protein kinase A, which phosphorylates cAMP response element-binding protein (CREB). The phosphorylated CREB binds to cAMP response element (CRE) of the DNA and recruits transcriptional co-activators, CBP (CREB-binding protein) and p300. This causes binding of RNA polymerase II and initiate transcription. At the end of transcription a single stranded mRNA is formed, which is translated into proteins that help to maintain synaptic plasticity, learning and memory, and modulate the brain’s reward circuit. Figure elements courtesy of Biorender (www.biorender.com) and Scidraw (www.scidraw.io); all remaining figures were created independently in Microsoft PowerPoint.

The development of addiction to BQ consumed with tobacco is attributable to several factors including nicotine dependence, neurochemical factors and psychological triggers (Benowitz, 2009; Papke et al., 2020b). Arecoline shares common receptors on the brain with nicotine, making BQ users more susceptible to nicotine addiction, and encouraging the addition of tobacco to BN preparations. This nicotine-like activity of arecoline causes physical dependence and withdrawal symptoms. Additionally, arecoline influences the brain’s neuronal receptor system, particularly the ones targeted by narcotic substances, namely, the nicotinic acetylcholine receptors (nAChRs) (Figure 2), and provoke addiction (Chatterjee and Gupte, 2023). The α4β2* subtype of nAChRs is believed to be the primary receptor driving nicotine dependence in humans (Benowitz, 2009). Nicotine binds specifically to nicotinic acetylcholine receptors (nAChRs) to provoke addiction. This binding causes receptor activation, and an influx of Na^+^ and Ca^2+^ ions into the neuroplasm. The action potential causes neuronal depolarization and the release of neurotransmitters in the synapse (George and Weinberger, 2008) (Figure 2). The release of neurotransmitters such as dopamine, glutamate, and gamma aminobutyric acid contribute to the development of nicotine dependence. Additionally, nicotine withdrawal can be facilitated by means of the corticotropin-releasing factor. The shared connection between arecoline and nicotine via the nAChRs hints at the potential effectiveness of using partial receptor agonists for tobacco cessation in also helping individuals quit BQ with tobacco (Papke et al., 2015).

Some studies indicate that various forms of BN produce differing concentrations of arecoline. The arecoline concentration in mature nuts is roughly 0.09%, whereas unripe BN has a concentration of 0.14% (Jantarat et al., 2013). The highest levels of arecoline, approximately 1.15%, are typically found in raw BN. Similarly, the arecoline content in other BN-containing products, such as pan masala, boiled nuts, and roasted nuts is recorded at 0.94%, 0.79%, and 0.85%, respectively (IARC, 2021). Thus, it is rational to consider that the form of BN or type of tobacco product consumed may also contribute to BQ dependence. Furthermore, several studies have found that added flavourings can enhance the absorption of nicotine (Pickworth et al., 2014; Sharma et al., 2023), which may be attributed to the higher nicotine content in flavoured products. A similar mechanism may apply to arecoline, whereby the addition of BN/tobacco-based flavourings could enhance arecoline uptake across the oral mucosa.

## 5 Role of monoamine oxidases in nicotine dependence

Monoamine oxidases (*MAO*) are flavin-containing enzymes localized at the outer mitochondrial membrane (Lv et al., 2023). These enzymes catalyse the oxidative deamination of a diverse range of biogenic amines, including dopamine, serotonin, norepinephrine, epinephrine and other catecholamines. By regulating synaptic concentrations of these neurotransmitters, *MAO* enzymes play a critical role in regulating physiological functions such as mood, memory, locomotion, and arousal (Lewis et al., 2007; Roeder, 2016).

On the basis of substrate specificity and sensitivity to specific inhibitors, *MAO* can be subdivided into two isoforms - *MAO-A* and *MAO-B.* The 15 exons that make up the *MAO-A* and *MAO-B* genes have a similar exon-intron structure. They are arranged in the opposite orientation, tail to tail, and separated by 24 kb on the X-chromosome. The transcription factor Sp1 controls both GC-rich promoters, but their characteristics are very different. The TATA box is present in the *MAO-B* gene but not in *MAO-A*. The intestine has the highest levels of *MAO-A* and *B*, which are co-expressed in the majority of human tissues. However, only *MAO-A* is expressed by the placenta and fibroblasts, while only *MAO-B* is expressed by platelets and lymphocytes (Shih and Chen, 2004).

Additionally, the two isoforms share 70% of identical polypeptide sequence, with comparable molecular weights of 59 kDa and 58 kDa, respectively (Shih et al., 1999). Each isoform performs distinct functions and are located at specific neuronal sites. The *MAO-A*, principally found in DAergic axon terminals supports the metabolism of neurotransmitters such as norepinephrine, epinephrine, serotonin, and melatonin. In contrast, *MAO-B* is limited to the breakdown of phenylethylamine and benzylamine, and is mainly present in astrocytes and serotonergic neuron (Cho et al., 2021).

Although the role of *MAO* inhibitors in tobacco smoke and their contribution to smoking addiction is well-documented, much less is known about the impact of these inhibitors in smokeless tobacco. Hogg (2016) proposed that several chemicals in tobacco might inhibit *MAO* in smokers through additive or combined effects. Tobacco smoke contains several recognized *MAO* inhibitors, such as β-carbolines, farnesyl acetone, and tetrahydroisoquinolines, (Hogg, 2016; Hong et al., 2022a), among which the β-carbolines harman and norharman are recognised as the principal constituents within tobacco smoke associated with *MAO* inhibition (George and Weinberger, 2008). By blocking *MAO* activity, these compounds reduce the breakdown of neurotransmitters linked to the brain’s reward circuitry, particularly dopamine, hence amplifying the addictive potential of smoking. On the other hand, *MAO* enzymes regulate neurotransmitter homeostasis and directly modulate the strength of reward signaling by oxidatively deaminating and breaking down monoamines including serotonin, dopamine and epinephrine. *MAO-A* and *MAO-B* thus play significant roles in complex behaviours like drug use and personality traits, and are recognized as important drug targets. Blocking them can influence temperament and have been utilized to treat conditions including depression, social anxiety, and nervousness (Hong et al., 2022a).

Smith et al. (2016) reported that inhibiting *MAO-A,* rather than *MAO-B,* leads to an increased propensity to self-administer nicotine at minimum doses. This indicates that components in cigarette smoke inhibiting *MAO-A* may enhance nicotine’s fundamental rewarding effect and its ability to amplify other reinforcing stimuli (Hong et al., 2022b). Investigation of Swedish snus, a form of smokeless tobacco (SLT), revealed that its aqueous extracts consistently exhibited robust inhibition of both *MAO-A* and *MAO-B*. However, snus can inhibit *MAO* at higher concentrations than smoking tobacco. Additionally, snus products are more addictive than the heated tobacco products or e-cigarettes. This is because *MAO* inhibitors are retained within the tobacco product itself but are not transferred into the aerosol during heating process used in e-cigarettes. This difference in chemical transfer could impact their relative potential to reinforce dependence beyond nicotine alone (van der Toorn et al., 2019).

## 6 Monoamine oxidases and BQ addiction

Arecoline is reported to have properties similar to those of *MAO-A* inhibitors (Hung et al., 2024; Ko et al., 2020), as it downregulates *MAO-A* mRNA and protein expression. This downregulation increases synaptic serotonin and dopamine levels, potentially contributing to antidepressant effects and dependence (Ko et al., 2020). Chen et al. (2012), found that arecoline reduced *MAO-A* mRNA and protein expressions at 100–200 μg/mL in SH-SY5Y cells (*in vitro*) and in rat whole brains at days 30–45 (*in vivo*), rather than directly inhibiting MAO- A enzyme activity. As a consequence, IC_50_ and Ki values, which are typically obtained during inhibition of enzyme activity cannot be determined in response to arecoline. In addition, they also observed that human plasma *MAO-A* activity was positively correlated with BQ exposure in a cohort of 1,307 Taiwanese aborigines, with genetically predisposed individuals carrying the risk alleles of *MAO-A* exhibiting lower, albeit statistically non-significant levels of MAO-A (Chen et al., 2012).

Thus, it is likely that chronic and simultaneous exposure to BN, may develop selective tolerance (neuroadaptation) which perpetuate cycles of dependency (Ko et al., 2020). Moreover, antidepressants such as selective serotonin reuptake inhibitors (SSRIs) and *MAO-A* inhibitors have been reported to reduce the severity of BQ addiction and the amount of BQ consumed (Hung et al., 2024), suggesting a significant role of *MAO-A* in BQ addiction (Hung et al., 2024). Furthermore, Taiwanese males with SNPs rs2283725 and rs5953210, and females with SNPs rs2283725 and rs5953210 in the *MAO-A* gene were found to be predisposed to heavy BQ use (Chen et al., 2012). The SNP rs5953210 variation of the *MAO-A* gene was reported to determine addiction severity through its effects on neurotransmitter metabolism, particularly dopamine and serotonin, which play vital functions in the regulation of mood and reward circuits (Hung et al., 2024).

While *MAO-A* inhibition provides a mechanistic link, it is unlikely to fully explain the heightened dependence observed in the combined users of BQ and tobacco. Both arecoline and nicotine can be rapidly absorbed through the oral mucosa, particularly in the presence of slaked lime, which increases oral pH and facilitates uptake (Pickworth et al., 2014; Bose et al., 2023; Sharma et al., 2023). BQ prepared with the inflorescence of the betel vine and slaked lime [Ca(OH)_2_] produces higher amounts of reactive oxygen species (ROS) than BQ made using betel piper leaves and BN (Bose et al., 2023). Additional elements that may affect nicotine absorption include local blood circulation, the hygroscopicity of the product, the size and surface area of the tobacco mixture, the buffering capacity to maintain pH, and the nicotine content of the tobacco. Several studies indicate that mint flavourings, such as menthol and wintergreen, can enhance nicotine absorption (Pickworth et al., 2014; Sharma et al., 2023), which may be attributed to the higher nicotine and/or arecoline content in the flavoured products. These findings imply that the combined use of BQ, tobacco, slaked lime, and other commercial flavouring substances might accelerate reinforcement more than BQ on its own. Thus, BQ addiction emanates from several overlapping biological processes, shaped further by the specific type and preparation of BQ consumed.

In one study, the dichloromethane fraction of areca nut was found to inhibit MAO-A isolated from rat brain with a high IC50 value of 665 ± 65.1 μg/mL, and produced responses similar to those of the selective pharmacological inhibitor of MAO-A, moclobemide, in pharmacological models of depression, though the direct inhibitory effects of areca alkaloids on MAO-A activity were not observed (Dar and Khatoon, 2000). These findings suggest that components of areca nut other than its alkaloids may be responsible for directly inhibiting MAO-A, which requires further investigation. Furthermore, comprehending the function of *MAO-A* may shed light on the mechanism of BQ addiction and aid in the discovery of putative biomarkers for the diagnosis and evaluation of BQ addiction (Hung et al., 2024), as well as help in the design of *MAO* inhibitors which may aid in the pharmacological management of BQ addiction.

## 7 Monoamine oxidase inhibitors in treating nicotine dependence

The *MAO* inhibitors are a chemically diverse group of 38 known compounds categorised into nine structural groups. Recently, five additional groups, namely, phenols, phenolic acids, coumarins, flavonoids and azaarenes have been identified as *MAO* inhibitors in tobacco and tobacco smoke. SLT and smoking tobacco comprises non-nicotinic components such as harman, trans-farnesol, menadione (2-methyl-1, 4-naphthoquinone) and kaempferol, all of which exhibit *MAO* inhibition property. While most phenols and phenolic acids acts as reversible and selective inhibitors of *MAO-A,* other components (such as trans-farnesol, menadione, and diosmein) selectively and reversibly inhibit *MAO-B* (Hong et al., 2022b). Among these, phenelzine, tranylcypromine and clorgyline are irreversible *MAO-A* inhibitors, while moclobemide is a reversible *MAO-A* inhibitor. In contrast, selegine, paragyline and rasagiline serve as irreversible *MAO-B* inhibitors, whereas labazemide is a reversible inhibitor.

Phenelzine, a non-selective *MAO* inhibitor, has shown potential in treating nicotine addiction in patients who did not respond to previous nicotine replacement therapies (NRT). Each of the patients showed a reduction in nicotine dependence as measured by the Fagerstörm Test for Nicotine Dependence (FTND) and Quality of Life and Satisfaction Questionnaire (Q-LES-Q-SF) (Curran et al., 2018).

Selegiline, which inhibits *MAO*-*B*, and moclobemide which targets *MAO-A*, have also demonstrated efficacy in raising abstinence rates and decreasing cravings in smokers (George and Weinberger, 2008). However, a study in rats found that *MAO* inhibitors present in tobacco smoke could work in synergy with nicotine to heighten its reinforcing properties. The inhibition of *MAO* activity was found to substantially increase the behavioural increase towards nicotine self-administration in rats, suggesting that *MAO* inhibitors enhance the addictive potential of nicotine. Thus, chronic exposure to *MAO* inhibitor treatment may lead to increased nicotine self-administration and sustained addiction (Guillem et al., 2005).

Clorgyline is a selective and irreversible *MAO-A* inhibitor which also increases self-administration of low-dose nicotine in rats. It mimics the *MAO-A* inhibiting compounds in cigarette smoke, and may play a key role in sustaining tobacco addiction, particularly at low nicotine doses. In contrast, rasagiline, a selective *MAO*-*B* inhibitor, did not increase self-administration, suggesting that *MAO-B* inhibition alone is insufficient to affect nicotine reward. Interestingly, tranylcypromine (TCP), a non-selective *MAO* inhibitor that blocks both *MAO-A* and *MAO-B* has increased sensitivity to nicotine. Even partial *MAO-A* inhibition, similar to the level found in chronic smokers (∼28%–43%), was enough to enhance low-dose nicotine self-administration. This means that not much *MAO*-*A* inhibition is required to strengthen nicotine’s effects (Smith et al., 2016).

The above findings emphasize that nicotine addiction is not only driven by nicotine alone, but *MAO-*inhibiting compounds present in tobacco products (both smoking and smokeless), especially those that suppress *MAO-A*. Thus, short-term and controlled doses of *MAO* inhibitors may support cessation when timed and prescribed appropriately, but chronic and unregulated *MAO* inhibition may enhance the reinforcing effects of nicotine, emphasising the requirement for a supervised cessation approach.

## 8 *MAO* inhibitors in BQ cessation therapy

Since arecoline and nicotine share similar pathways of action, and *MAO-A* inhibitors have significant impact on addiction to both BQ and tobacco products, it is hypothesised that *MAO* inhibitors might be valuable in BQ cessation strategies, similar to their role in nicotine addiction treatment. By modulating *MAO* activity, these inhibitors could help alleviate withdrawal symptoms and cravings associated with arecoline dependence, much like their effects in NRT (Hong et al., 2022b).

BQ, through its active component arecoline, downregulates *MAO-A* expression at both the transcriptional and protein levels (Chen et al., 2012; Ko et al., 2020; Yan et al., 2024). Prolonged BQ exposure induces neuroadaptation to this effect (Shao et al., 2026). Following cessation, the loss of arecoline-mediated *MAO-A* inhibitory-like properties, together with the gradual normalization of *MAO-A* expression may contribute to alterations in neurotransmitter homeostasis. Such changes may lead to the development of withdrawal symptoms, frequently triggering cravings and relapse, although the precise temporal relationship between *MAO-A* recovery and withdrawal remains to be fully elucidated. In this context, reversible monoamine oxidase inhibitors, such as moclobemide, may attenuate these withdrawal effects by directly inhibiting *MAO-A* enzyme activity (Ahmadi, 2026), thereby increasing synaptic concentrations of dopamine and serotonin. This may provide selective, controlled, and predictable *MAO-A* inhibition, enabling a gradual reduction in dosage of BQ use to restore normal synaptic function. In contrast to arecoline’s variable inhibition, which depends on chewing frequency, moclobemide may maintain stable neurotransmitter levels during cessation, thereby reducing withdrawal symptoms and cravings, and supporting sustained abstinence.

For BQ cessation strategies, the reversible inhibitors are preferred over irreversible ones due to the dietary restrictions associated with the latter, such as the well-known “Cheese effect”, which results from the accumulation of tyramine when *MAO-A* is permanently inhibited (George and Weinberger, 2008). Beyond the “Cheese effect”, *MAO* inhibitors carry additional safety concerns that warrant consideration if they were to be applied in BQ cessation therapy. These include serotonin syndrome, a rare but potentially life-threatening condition that may occur when *MAO* inhibitors are combined with other serotonergic agents, such as antidepressants, certain analgesics like tramadol or meperidine, or herbal supplements including St. John’s wort. Sudden discontinuation of *MAO* inhibitors can also result in withdrawal symptoms, including dizziness, irritability, nausea, anxiety and insomnia. Gradual dose tapering is therefore recommended to minimize these risks. In addition, seizures have been reported both in cases of overdose and following withdrawal from *MAO* inhibitors, particularly when misuse is involved (Patel and Saadabadi, 2025).

**FIGURE 3:**
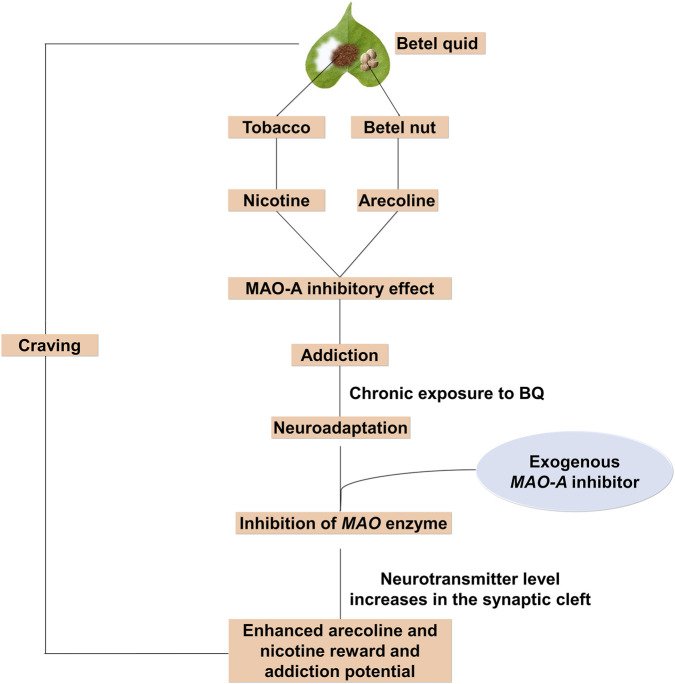
Role of *MAO* inhibition in BQ addiction. BQ chewing exerts cumulative *MAO-A* inhibitory effects due combined actions of arecoline and nicotine, which contributes to addiction and neuroadaptation in chronic BQ users. Prolonged exposure of exogenous *MAO-A* inhibitors for can sustain activation of the brain’s reward circuitry, leading to persistent craving and dependency. Figure elements courtesy of Biorender (www.biorender.com); all remaining figures were created independently in Microsoft PowerPoint.

**FIGURE 4:**
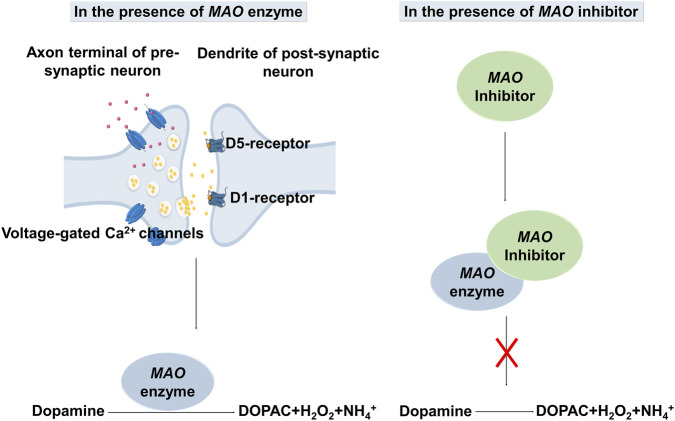
Mechanism of dopamine regulation in the presence and absence of *MAO*-inhibitors. In the presence of the *MAO* enzyme, dopamine is broken down into 3,4-dihydroxyphenylacetic acid (DOPAC), hydrogen peroxide (H_2_O_2_) and ammonium ion (NH_4_^+^). However, when an *MAO* inhibitor (e.g., moclobemide) is present, it inhibits the *MAO* enzyme from breaking down neurotransmitters present in the synapse. As a result, dopamine concentration increases, helping to reduce cravings and withdrawal symptoms. Figure elements courtesy of Biorender (www.biorender.com); all remaining figures were created independently in Microsoft PowerPoint.

Since some BQ chewers mix tobacco with their quid, it is plausible that they are exposed to non-nicotinic *MAO* inhibitors present in tobacco. Additionally, arecoline itself acts as an *MAO* inhibitor which further contributes to the regulation of *MAO* activity. This suggests that users who consume BQ with tobacco may experience combined effects from both arecoline and tobacco-derived *MAO* inhibitors, which could significantly influence addiction mechanisms and cessation strategies (Figure 3). *MAO-A* inhibition enhances dopamine-mediated reward effects, making cessation therapies more effective by reducing cravings and withdrawal symptoms (Figure 4). Therefore, individuals addicted to BN with tobacco mixtures may need more carefully controlled dosing strategies than those used for BN-only and tobacco-only users for effective addiction cessation. Furthermore, more studies are required to fully understand the ideal dosages and the long term effects of combining *MAO-A* inhibitors with nicotine or arecoline replacement therapies (ART). This could help establish guidelines for their use in treating nicotine and BQ addiction safely and effectively (Hong et al., 2022a; Laban and Saadabadi, 2023).

## 9 Natural *MAO-A* inhibitors and their potential role in BQ addiction

There is evidence that natural *MAO* inhibitors have a selective effect on patients with atypical depression. Compared to conventional *MAO* inhibitor drugs, natural and selective inhibitors may offer a safer therapeutic profile. Additionally, since *MAO* inhibitors are known to be effective in managing conditions such as depression, hypertension, neurodegenerative disorders, and even type 1 diabetes (Emory and Mizrahi, 2016; Chaurasiya et al., 2022), their use in individuals who are addicted to BQ or tobacco-based substances, could potentially provide cumulative therapeutic benefits. Natural *MAO* inhibitors present a dual-action therapeutic strategy for BQ addiction management. These compounds can simultaneously modulate addiction pathways while addressing comorbid conditions that frequently accompany substance use disorders.

Various types of naturally occurring compounds such as alkaloids, flavonoids (Chaurasiya et al., 2022), coumarins (Liang et al., 2021), xanthones (El Masaoudy et al., 2024; Oriola and Kar, 2024), anthraquinones (Paudel et al., 2019), chromenones (Agarwal et al., 2024), phenols and bromophenols (Bhuia et al., 2023; Dong et al., 2021; Irfan et al., 2022) may have the ability to counteract the neurochemical effects of BQ use and oral discomfort that commonly accompany BQ chewing, due to their well-documented effects on *MAO-A* inhibition (Agarwal et al., 2024; Chaurasiya et al., 2022) and neuroprotective property (Liang et al., 2021; Song et al., 2021; Talebi et al., 2021). Alkaloids such as harmane, norharmane, and tetrahydroharmine show strong *MAO-A* inhibitory properties, and have been shown to regulate DAergic and serotonergic pathways that are readily associated with addiction reinforcement, and induce anxiolytic, antidepressant and neuroprotective effects that help manage withdrawal symptoms and psychological stress associated with BQ cessation. Similarly, other compound classes including flavonoids, coumarins, xanthones, anthraquinones, chromenones, phenols and bromophenols, exhibit varying degrees of *MAO-A* inhibition alongside complementary therapeutic properties. These properties may offer an integrative approach in managing BQ-induced inflammation, psychological distress, blood-clot, tissue damage, sores and carcinogenesis. Agents with these protective properties can further strengthen cognitive function and support cessation by improving focus, memory, and impulse control, thereby empowering individuals to better resist cravings, adhere to behavioural interventions, and achieve long-term abstinence from BQ. Interestingly, glycitein, a flavone, possess estrogenic properties along with *MAO-A* inhibitory effect (Prajapati et al., 2021), which may be particularly beneficial for female BQ users, though further investigation regarding pregnancy safety is warranted.

While several natural compounds are endowed with *MAO-A* inhibitory property, their clinical applications are limited due to poor oral bioavailability, quick metabolism, and restricted blood-brain permeability. Such limitations may be circumvented by various advanced delivery systems including nano-encapsulation, phytosome complexes, and co-administration with bioavailability enhancers (Brown et al., 2022; Singh and Kumar, 2018). These strategies may confer metabolic stability and central nervous system (CNS) penetration to enable sustained inhibition of *MAO-A* necessary for modulating neurotransmitter pathways implicated in BQ abuse.

Thus, development of a systematic therapeutic strategy for BQ addiction may require identification and screening of *MAO-A* modulating bioactives through *in silico* approaches, followed by validation through *in vitro* and preclinical models of arecoline dependence. This may be complemented by formulation of user-centric delivery systems tailored to mimic the conditioned behavioural and sensory microenvironment associated with BQ use. However, comprehensive clinical evaluation remains imperative to substantiate safety, efficacy, and translational potential in BQ users.

## 10 Study limitations

The current literature review was restricted to PubMed and Google Scholar and included only English-language publications. To minimize the risk of omitting relevant studies, backward citation searching, forward citation tracking, and targeted supplementary searches were conducted.

Studies on the mechanisms of addiction to BQ have largely focused on the role of arecoline, and there are no published IC_50_ or Kᵢ values with respect to the plausible inhibitory role exerted by arecoline on MAO-A enzyme activity. Indeed, the effect of arecoline on MAO-A activity appears to be largely mediated by downregulation of gene expression (transcriptional regulation) in genetically predisposed individuals, which differs mechanistically from the competitive or noncompetitive enzyme inhibition characterized by IC_50_ or Kᵢ constants, as seen with established *MAO* inhibitors such as moclobemide (reversible, Kᵢ approximately 10–50 nM). In the absence of direct enzymatic inhibition data, the pharmacological relevance of arecoline’s *MAO-A* inhibitory activity at typical BQ consumption levels remains unclear. Moreover, studies investigating the role of other constituents of areca nut in this context are limited, thereby further restricting in depth mechanistic insights.

Arecoline primarily acts as a muscarinic acetylcholine receptor agonist (M1–M4) (Shao et al., 2026). *MAO* inhibition is unlikely to be the principal mechanism underlying its addictive potential. BQ also contains additional alkaloids, such as arecaidine, guvacine, and guvacoline, and exhibits several pharmacological effects (Sharan et al., 2012). These include dopamine modulation, reduction of noradrenaline, and GABAergic activity. *MAO* inhibition alone does not fully explain the addictive properties of BQ (Shao et al., 2026), and targeting *MAO-A* may address only a single aspect of BQ dependence. Furthermore, individuals who chew BQ mixed with tobacco are exposed to non-nicotinic *MAO* inhibitors present in tobacco smoke, which may lead to combined pharmacological effects that are not well understood and may necessitate distinct dosing strategies compared to BQ-only or tobacco-only users.

Additionally, no clinical trials have delved into the use of *MAO* inhibitors specifically for BQ cessation. Consequently, optimal dosing strategies for reversible versus irreversible *MAO* inhibitors in populations remain entirely undefined. Moreover, no natural *MAO-A* inhibitors have been evaluated in clinical trials for the treatment of BQ addiction, and the safety profiles of these inhibitors in BQ users with comorbid conditions or associated toxicities have not been established.

The optimal dosages and long-term effects of combining *MAO-A* inhibitors with NRT or ART have not been investigated. Individuals’ dependent on BQ-tobacco mixtures may be exposed to synergistic or antagonistic interactions among multiple *MAO* inhibitors, including arecoline, tobacco-derived *MAO* inhibitors, and therapeutic *MAO* inhibitors. Currently, there are no established guidelines for the safe combination of *MAO-A* inhibitors with other cessation pharmacotherapies. Additionally, genetic polymorphisms of *MAO-A* may affect individual vulnerability to BQ dependence and therapeutic response to *MAO* inhibitor treatment, however, no studies have stratified BQ cessation outcomes by *MAO-A* genotype.

## 11 Importance and future perspectives

BQ chewing, its dependence, and treatment remain strikingly understudied despite its global prevalence. The issue is of grave concern given the significant implications of long term BQ usage on human health. Effective cessation strategies are thus the need of the hour as preventive measures against the harmful consequences of BQ use. The interrelation of BQ dependence with *MAO-A* activity represents a promising, yet unexplored domain that may potentially elucidate the underlying interaction between arecoline’s *MAO-A* inhibitory properties and the brain’s reward circuitry and mood regulation, serving as a target for pharmacological cessation interventions. The complexity of the issue further increases for users who combine BQ with tobacco, creating a dual exposure to *MAO* inhibitors from areca nut and tobacco, that may intensify addiction through synergistic neurochemical mechanisms. Moreover, variations in the form of BN and in the type of processed or unprocessed tobacco used may further contribute to dependence by altering the arecoline and/or nicotine concentration in the mixture, thereby amplifying the overall *MAO* inhibitory activity of BQ. Thus, research investigating the combined effects of arecoline, tobacco-derived *MAO* inhibitors, nicotine, and cessation pharmacotherapies will potentially unveil the synergistic, additive, or antagonistic interactions that can be used to develop suitable dosing strategies. However, critical evaluation is required to assess the risk of adverse effects of these drugs, such as serotonin syndrome, hypertensive crisis, and other drug-drug interactions when *MAO* inhibitors are administered alongside NRT, antidepressants, or other cessation medications, as well as patients suffering from underlying comorbidities.

Current evidence indicates that arecoline downregulates *MAO-A* mRNA and protein expression, however, the precise mechanism underlying direct enzyme inhibition by arecoline or its binding to *MAO-A*, remains unclear. Studies employing human *MAO-A* enzyme assays are necessary to determine whether arecoline functions as a competitive, noncompetitive, or allosteric inhibitor, and to establish pharmacological parameters such as IC_50_, Kᵢ, and receptor-binding affinities. Additionally, pharmacokinetic-pharmacodynamic investigations should assess the arecoline concentrations absorbed during typical BQ consumption that are sufficient to induce *MAO-A* inhibitory effects in humans.

Furthermore, randomized controlled trials should evaluate both synthetic and naturally derived *MAO* inhibitors, with rigorous assessment of efficacy, safety, tolerability, and relapse prevention.

Developing precise biomarkers based on *MAO-A* expression patterns could also help in early diagnosis and in assessing BQ addiction severity. New drugs that target *MAO-A* pathways may open different avenues for treatment. Carefully timed and dosed *MAO-A* inhibitors could support BQ/BN cessation rather than reinforcement. These therapeutic treatments need to be designed around cultural awareness, acknowledging BQ’s social and cultural significance across communities.

Moreover, future research focusing on multiple targets associated with BQ use, including multi-omics methods such as transcriptomics, proteomics, and metabolomics, could elucidate how cholinergic, DAergic, noradrenergic, GABAergic, and monoaminergic pathways interact in BQ addiction. Furthermore, by exploring the multifaceted neurological relationships among BQ’s components (including arecoline, arecaidine, guvacine, guvacoline, and other alkaloids), researchers could potentially highlight key mechanisms underlying BQ addiction.

The safety of *MAO*-targeted interventions should be systematically evaluated in BQ users with prevalent comorbidities, such as oral submucous fibrosis, oral potentially malignant disorders, hepatotoxicity, and cardiovascular disease. Long-term observational studies and clinical trials are essential to establish comprehensive safety profiles in these vulnerable populations.

Precision medicine approaches constitute a promising avenue for future research. Subsequent studies should examine the impact of *MAO-A* genetic polymorphisms and other genetic determinants of neurotransmitter metabolism on susceptibility to BQ dependence, withdrawal severity, and treatment response. Stratifying participants by genotype may facilitate the identification of subgroups most likely to benefit from *MAO*-targeted therapies and inform the development of personalized cessation strategies.

Translating biological findings into real-world applications necessitates aligning research evidence with community-based interventions. This can begin with the integration of *MAO-A* targeted therapy with the established WHO’s guidelines, including BQ cessation into tobacco dependence programs. Digital health technologies may further provide an opportunity to expand access to treatment for rural communities where BQ use is widespread, but often overlooked.

Ultimately, effective diminution of this global public health concern will necessitate a concerted effort that integrates biological research, pharmacological innovation, and culturally appropriate implementation strategies, thereby offering a comprehensive approach that addresses both the molecular and social determinants of BQ addiction.

## 12 Conclusion

BQ cessation and interventions warrant a comprehensive critical evaluation, as they are not localized issues but pressing global health priorities affecting millions worldwide. In this literature review, the neurocognitive aspects of *MAO-A* and *MAO* inhibitors have been identified as a promising target for understanding BQ addiction and developing effective interventions. Significantly, the interaction between *MAO-A* inhibition and reward pathways offers a more comprehensive view of BQ’s addictive properties, particularly when co-used with tobacco. Existing knowledge about arecoline’s interaction with *MAO-A* remains limited, focusing primarily on *MAO-A’s* downregulation through arecoline rather than on direct enzyme inhibition. Current cessation strategies are primarily educational and behavioral, with limited pharmacological interventions, underscoring a significant treatment gap for the world’s fourth most commonly abused substance. Future research should elucidate the molecular mechanisms underlying BQ dependence, optimize *MAO-A* modulation dosing strategies with respect to natural inhibitors to address BQ addiction. Consequently, effective BQ cessation requires a comprehensive understanding and multidisciplinary approaches that integrate both biological and sociocultural factors.
